# Die for the community: an overview of programmed cell death in bacteria

**DOI:** 10.1038/cddis.2014.570

**Published:** 2015-01-22

**Authors:** N Allocati, M Masulli, C Di Ilio, V De Laurenzi

**Affiliations:** 1 Department of Experimental and Clinical Sciences, University “G. d'Annunzio”, Chieti I-66013, Italy; 2 Center of Excellence on Aging (Ce.S.I.), University “G. d'Annunzio”, Chieti I-66013, Italy; 3 BIOUNIVERSA srl, University of Salerno, Fisciano (SA), Italy

## Abstract

Programmed cell death is a process known to have a crucial role in many aspects of eukaryotes physiology and is clearly essential to their life. As a consequence, the underlying molecular mechanisms have been extensively studied in eukaryotes and we now know that different signalling pathways leading to functionally and morphologically different forms of death exist in these organisms. Similarly, mono-cellular organism can activate signalling pathways leading to death of a number of cells within a colony. The reason why a single-cell organism would activate a program leading to its death is apparently counterintuitive and probably for this reason cell death in prokaryotes has received a lot less attention in the past years. However, as summarized in this review there are many reasons leading to prokaryotic cell death, for the benefit of the colony. Indeed, single-celled organism can greatly benefit from multicellular organization. Within this forms of organization, regulation of death becomes an important issue, contributing to important processes such as: stress response, development, genetic transformation, and biofilm formation.

## Facts

- Programmed cell death (PCD) is not restricted to multicellular organisms.
- Different forms of death have been described in bacteria.
- Bacteria are able to induce cell death in a part of the population in response to adverse conditions to favour the survival of the community.
- PCD has a crucial role in the development of a bacterial population.

## Open Questions

- Do bacterial and eukaryotic PCD have a common origin?
- Are there additional unknown forms of death in prokaryotes?
- Can we exploit knowledge on molecular mechanisms of bacterial PCD to develop alternative therapeutic strategies against multiresistant pathogens?

In eukaryotes, death is essential for life. Indeed, PCD is fundamental for many processes, including: embryogenesis, tissue homeostasis, and immune and stress response.^1, 2, 3, 4^ Therefore it has been assumed that PCD had emerged with multicellularity, being absent in unicellular organisms.^3, 5, 6^ However, it is now clear that PCD also occurs in unicellular eukaryotes and bacteria and is therefore present in all life kingdoms.^3, 7, 8, 9, 10, 11, 12, 13, 14^ Microorganisms are social organisms and many of them can switch from unicellular to multicellular organization such as microbial colonies, biofilms, and aggregates.^15^ Multicellular organization offers many advantages, such as increased protection against hostile environments, increased genetic diversity, and improved food availability.^16, 17, 18^ A bacterial population – acting as a multicellular organism – would use PCD programmes to sacrifice part of the colony to sustain the survival of the remaining cells.^7, 19^

Bacteria communicate through a process named quorum sensing (QS) to modulate gene expression in response to changes in cell density.^20^ The increase of cell density, resulting in the accumulation of the signalling molecules in the environment, activates the response. QS allows bacteria to synchronize the gene expression pattern of the colony and thus behave like a multicellular organism and is involved in several bacterial processes such as biofilm, sporulation, production of virulence factors, and competence for DNA uptake.^20, 21, 22^ Clearly QS may result in activation of death pathways to control colony size as shown by some examples discussed in this review.

In multicellular eukaryotes, death comes in many flavours and the term PCD describes a number of biological processes that differ for morphological characteristics as well as for the underlying molecular pathways. Many of these have now been well characterized at the molecular level, and recently, the Nomenclature Committee on Cell Death has proposed a new classification based on biochemical features rather than morphological characteristics.^23, 24, 25, 26^

Similarly, different forms of PCD can be observed in prokaryotes but these are in general less characterized.^7, 27, 28, 29, 30^ As an example, PCD has been demonstrated experimentally by synthetic-biology approaches in *E. coli* cells, outlining the benefits of altruistic death under stressing conditions.^31^

Bacteria are able to control cell death under several stressful conditions, such as high temperature, amino-acid starvation, and antibiotic treatment.^29, 32, 33^ Indeed, the well-known toxin–antitoxin (TA) system has a role in cell survival under cellular damage or several stressing conditions, resulting in either cell growth arrest or a form of cell death similar to apoptosis.^29^ Furthermore, it appears that PCD has a key role in various developmental processes, such as: autolysis of bacterial cells during the formation of fruiting body in myxobacteria,^34^ hyphae differentiation in streptomyces,^35^ sporulation of bacilli, and DNA transformation in streptococci.^36^

In this review, we try to give a comprehensive overview of the different forms of death described in bacteria.

## PCD in Stress Response

A bacterial community can induce death in a part of the population in response to various stress conditions to favour the survival of the colony, including: oxidative stress, radiation exposure, nutrient deprivation, phage infections, and many others. In most of these cases, PCD is induced through TA mechanisms.

### TA systems

TA systems are involved in several processes, such as formation of persistent cells, plasmid stabilization, peptidoglycan (PG) synthesis, resistance to bacteriophages and antibiotics, inhibition of macromolecule, and biofilm formation.^29, 37, 38^ Most bacteria and archaea contain TA loci in their genomes, often in multiple copies, present in both extra chromosomal and chromosomal DNA.^29^ TA loci are typically organized in operons with two genes constitutively encoding a stable toxin that disrupts an essential cellular process and an unstable antitoxin that prevents its toxicity.^29, 32, 39^ Toxins and antitoxins form a stable complex inhibiting the toxin activity under normal growth conditions. In case of cellular damage or stress conditions, antitoxins are degraded, allowing the free toxin to bind its cellular target. To date, three types of TA systems are known and are classified on the basis of the function of the antitoxin and the composition of the TA system.^29, 32, 39^ More recently, two additional types of TA systems have been identified;^32, 40^ however, it is still questionable if their distinguishing features justify a new classification.

**Figure 1:**
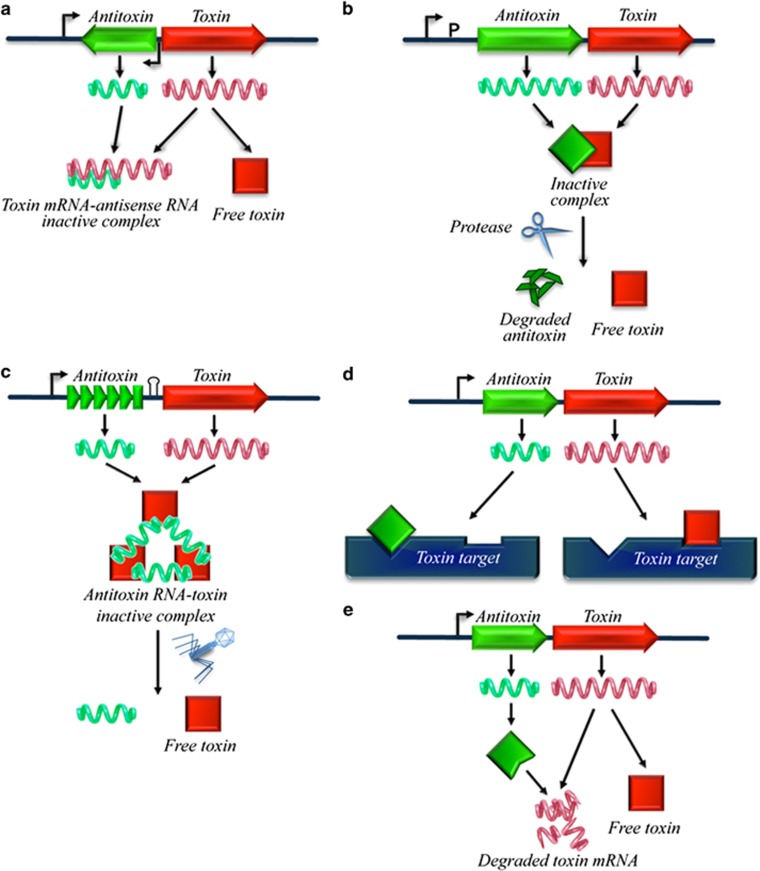
Bacterial TA systems. Bacterial TA systems are composed of a toxin and an antitoxin that neutralizes its effect. They are classified on the basis of the function of the antitoxin and the composition of the TA module. In all the TA system, in response to various stimuli the antitoxin is degraded allowing the toxin to act on its target generally resulting in either bacterial growth arrest or cell death. (**a**) Type I: antisense RNA antitoxin binds to the mRNA encoding for the toxin blocking its translation. Loss of the unstable antisense mRNA allows transcription of the sense strand. (**b**) Type II: toxin and antitoxin generally transcribed in the same operon form an inactive complex. Protease-dependent degradation of the antitoxin in response to stress frees the active toxin. (**c**) Type III: antitoxin RNA binds and inactivates to the toxin protein. (**d**) Type IV: the antitoxin prevents the effect of the toxin by binding the toxin target. Again in response to stress, degradation of the antitoxin allows binding of the toxin to its target. (**e**) Type V: the antitoxin binds and cleaves the mRNA encoding for the toxin

In type I TA systems, toxin gene expression is regulated by an antisense RNA (RNA-antitoxin) transcribed from a gene adjacent to the toxin gene but in reverse orientation.^29^ Type I TA systems – that is, the *hok/sok* system – mediate plasmid maintenance, inducing death in plasmid-free cells.^41, 42^ Annealing of the antisense RNA to the toxin mRNA prevents toxin translation (Figure 1a). When the plasmid is lost following cell division, the antitoxin (an unstable RNA) is rapidly degraded allowing translation of the more stable toxin mRNA thus leading to cell death. The molecular mechanism through which these toxins kill the cell is still unclear but it is probably associated with membrane depolarization and increased membrane permeability.^41, 42^

In type II TA systems, two genes, codifying for two small proteins, are organized in an operon and are regulated at the transcriptional level. The two proteins form an TA stable complex that inhibits the harmful effects of the toxin^29^ (Figure 1b). Following cell damage, stress-induced proteases digest the antitoxin freeing the toxin leading to inhibition of cell growth or to cell death.^29^ Several type II TA systems have been identified and their biological role characterized.^37, 43, 44^ The first type II TA system described was the *mazE/mazF* module that is extensively diffused among bacteria and is based on the activity of the toxin MazF, a ribosome-independent mRNA interferase.^45^ This enzyme is a specific endoribonuclease that, when induced, cleaves cellular mRNAs inhibiting cell growth. In *E. coli*, the toxin is activated in response to several types of stress conditions, such as: high temperature, amino-acid starvation, oxidative stress, and antibacterial pressure, resulting in cell growth arrest and eventually in PCD.^37, 46^ Antibacterials that inhibit RNA and/or protein synthesis affect *mazEF* expression.^19^ The consequent drastic reduction of cellular concentration of MazE releases the MazF toxin triggering cell death.^19^ The *mazEF* module is also involved in bacterial persistence (see below). In *Myxococcus xanthus*, an MazF homologue has a crucial role in the fruiting body formation (see below). It has been recently reported that *mazEF*-mediated cell death in *E. coli* is a population phenomenon requiring the presence of a QS factor called extracellular death factor (EDF).^47^ EDF is a linear pentapeptide that specifically affects the toxin significantly amplifying its enzymatic activity. Similar results have been obtained for the ChpBK toxin of the type II TA system *chpBIK* in *E. coli*.^47^ More recently, EDFs have been also found in the Gram-positive *Bacillus subtilis* and in the Gram-negative *Pseudomonas aeruginosa*.^48^ EDFs of *B. subtilis* and *P. aeruginosa* have been shown to be able to trigger *E. coli mazEF*, providing the first example of a QS factors participating in interspecies bacterial cell death.^48^ Therefore, it has been proposed that the induction of the altruistic suicide mechanism by EDFs may be used from a bacterial species – under stressing conditions – to kill another in a mixed population.^48^ EDFs have the potential to be exploited to generate a new class of antibiotics that trigger death from outside the bacterial cells.^49^

Members of the epsilon/zeta TA family are also involved in the virulence of several human pathogens.^50^ An example is the PezA/PezT system (*p*neumococcal *e*psilon *z*eta) in *Streptococcus pneumoniae*. The PezT toxin phosphorylates the PG precursor uridine diphosphate-*N*-acetylglucosamine, causing the inhibition of MurA. This enzyme catalyses one of the first steps of PG synthesis, therefore its block results in autolysis of rapidly growing bacteria. As a consequence, the pore-forming toxin pneumolysin, a major virulence factor that accelerates infection progression, is released.^50^

Other type II TA modules, that is, *hipBA*, *mazEF*, and *mqsRA*, are involved in bacterial persistence.^29, 51^ In this case, toxins, rather than causing cell death, induce a quasi-dormant cell state. This is characterized by the presence in a bacterial population of a small group of slowly growing cells – called persisters – highly tolerant to a number of environmental insults such as antibiotics, to which the numerically prevalent exponentially growing cells are sensitive.^51^ Usually this state is induced by toxins released by stress-induced proteases. One of these is the HipA (*hi*gh *p*ersistence) toxin which together with the corresponding HipB antitoxin is encoded by *hipBA* locus. Inhibition of cell growth and stimulation of persisters formation requires HipA serine kinase activity,^52^ which supposedly exerts its effect through phosphorylation and consequent inhibition of the elongation factor Tu (EF-Tu), essential for the elongation phase of protein synthesis.^52^ Finally, type II TA systems also have a role in biofilm formation as described later.^50, 53, 54^

Type III TA systems consist of an RNA antitoxin molecule that inhibits toxins by the formation of a RNA–protein complex.^55, 56^ One example is the ToxIN system, one of the many mechanisms that bacteria use to withstand bacteriophage infection.^57^ In this system, altruistic suicide of an infected cell reduces phage infection within the population. The abortive infection system, ToxIN, first identified in the phytopathogen *Erwinia carotovora* and later in several genera of Gram-negative and Gram-positive bacteria, functions as a typical type III TA system via a novel RNA–protein mechanism^55, 58, 59^ (Figure 1c). *toxN* gene – codifying an endoribonuclease – is preceded by a short palindromic repeat that acts as a transcriptional terminator (stem-loop), regulating the relative levels of both antitoxin RNA and toxin transcript. Upstream there is the antitoxin (ToxI), a repetitive array containing 5.5 tandem repeats of a 36 nt sequence. Each 36 nt ToxI RNA repeat can inhibit the activity of ToxN. ToxIN complex is composed of a heterohexameric triangular assembly of three ToxN proteins interlaced by three ToxI RNAs.^59^ ToxI RNA antitoxin interacts directly with ToxN inhibiting its toxic activity instead of preventing its expression. During phage infection, the ToxI:ToxN ratio changes, probably due to alterations in host transcription or translation or the degradation of bacterial DNA, resulting in the release of the active toxin that consequently cleaves cellular and phage RNAs.^55, 60^

In type IV TA systems, the antitoxin does not inhibit the toxin through direct binding but neutralizes its toxicity by stabilizing the toxin target proteins^61^ (Figure 1d). A prototype of these systems is the YeeU/YeeV (CbeA/CbtA) module, involved in the regulation of cytoskeletal proteins and as a consequence of cellular morphology changes and division. The toxin CbtA inhibits the polymerization of the cytoskeletal proteins MreB and FtsZ. The antitoxin YeeU causes the opposite effect, interacting with two proteins that promote the assembly of the filaments. Under stress conditions, YeeU is degraded allowing CbtA to bind its targets resulting in altered cytoskeleton and inhibition of cell division.^61^ Finally and more recently, type V systems have been described in which the antitoxin acts as an endoribonuclease that degrades the mRNA coding for toxin mRNA (Figure 1e). *E. coli* uses such a system, where endoribonuclease GhoS specifically degrades the mRNA coding for the membrane lytic peptide GhoT by cleaving its mRNA.^62^

### Holin–endolysin system

dsDNA bacteriophages induce cell death through a holin–endolysin system,^63, 64^ at the end of the lytic cycle, to release new bacteriophage particles. Holins are small proteins that accumulate in the host membrane where they induce hole formation allowing endolysins – enzymes with PG hydrolase activity – to reach and degrade the PG leading to cell lysis.^63^ Antiholins finely tune the system by inhibiting holins, thus regulating the accurate timing of endolysin release.

## Role of PCD in Bacterial Development

PCD is essential for the proper development of a bacterial population and contributes to it in many ways: providing nutrients to the sibling cells; releasing components of the biofilm matrix such as DNA; and promoting special aspects of the life cycle and of biofilm development.

### Cannibalism and autolysis during sporulation

**Figure 2:**
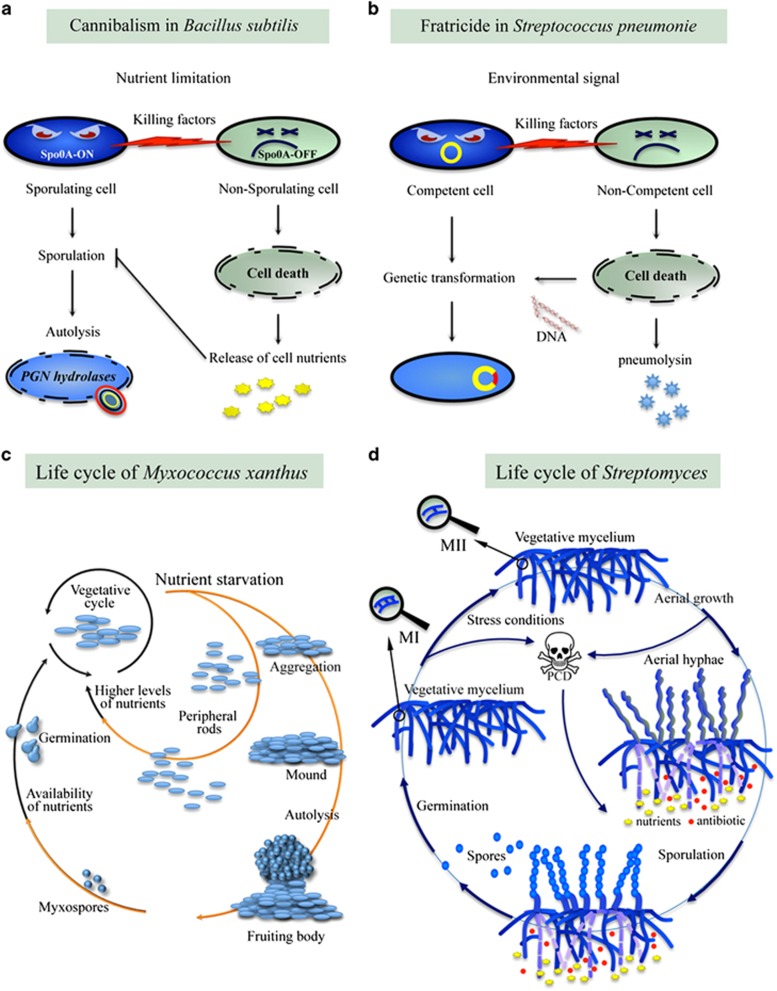
Examples of bacterial programmed cell death. (**a**) Upon nutrient limitation *Bacillus subtilis* undergoes two fates: sporulation or non-sporulation. Death of non-sporulating cells results in nutrient release that supports sporulation. Moreover, the mother cell in the sporulating population undergoes PCD to release the mature spore. (**b**) Competent *Streptococcus pneumonaie* cells induce death of non-competent cells in order to incorporate their DNA for genetic transformation. (**c**) Upon nutrient starvation of *Myxococcus xanthus*, while a small percentage of cells remains undifferentiated and forms the peripheral rods, the majority of cells undergoes fruiting body formation. During this process, a large number of cells is lysed in order to release nutrients for the remaining cells that will differentiate into mixospores. When nutrients are available, a new colony rises from proliferation of peripheral rods cells and germination of myxospores. (**d**) Under stressing conditions, a part of MI cells is subjected to PCD while the remaining viable cells differentiate into MII cells. In the second phase of PCD, a part of MII cells dies releasing nutrients to feed the aerial mycelium, which is developed from branches of the remaining viable cells of MII and rises above the surface. The apical cells of the aerial hyphae differentiate into spores that can spread in the environment

Endospores are metabolically dormant and resistant bodies, produced by *Bacillus* and *Clostridium* and other related species formed in response to high cell density and to severe external stress – such as nutritional stress for *Bacillus* spp. and acidification for *Clostridia* spp. Cell division during sporulation produces two different cells: a smaller one, the prespore which evolves into the spore and a bigger one, the mother cell, which is essential for spore formation (Figure 2a). When its maturation is completed the spore is released, and this requires PCD by autolysis of the mother cell. This phenomenon involves the activity of specific enzymes, PG hydrolases (see Box 1). In *Bacillus subtilis*, two PG hydrolases, LytC and CwlC, are present in large amounts and are responsible for the hydrolysis of the mother cell PG allowing the release of the mature endospore.^65^

During the early events of sporulation, before the process becomes irreversible, a part of *B. subtilis* cells can produce extracellular factors to induce a death program and ‘cannibalize' sister cells that release nutrients causing delay or complete block of the sporulation (Figure 2a). The process becomes irreversible when the cells committed to sporulate form an asymmetric polar septum.^66^

Cannibalism offers various advantages to the bacterial community. Sporulation process requires a lot of energy and time to be completed. Furthermore, in the availability of nutrients, spores do not return to active growth as efficiently as vegetative cells. Thus, in a mixed community, where most of the bacteria are non-sporulating, the germination of the spores could represent a disadvantage. In the absence of cannibalism, all bacteria sporulate at the same time, and therefore returning to vegetative life is slower and more complex. Therefore, cannibalism helps to maintain in the population a small percentage of spores and a higher number of growing cells, during the long-term stationary phase of growth.^66^ Moreover, in the mixed community where *B. subtilis* lives and competes with other species, bacteria not only gain nutritional benefits through consuming their siblings but also may eliminate potential competitors and predators.

The molecular mechanism of ‘cannibalism' involves two gene clusters, *skf* and *sdp*, controlled by the Spo0A regulon, the main regulator of sporulation.^36, 66^ Although the killing factor produced by *skfA-H* operon has not been completely characterized, it appears that its activity is similar to that of bacteriocins. In the second cluster of genes, the *sdpC* gene produces a peptide toxin, SDP, that acts on the proton motive force of the adjacent cells by inhibiting mobility and secretion of proteins and inducing cell lysis.^67^ SDP toxin appears to have a role also in the defence towards invading bacteria.^67^ In fact, the collapse of proton motive force inhibits flagellar motility in several species preventing their ability to invade the colony. Furthermore, it has been suggested that SDP can inhibit biofilm formation by surrounding bacterial species, decreasing competition in the ecological niche. Finally, bacteria with compromised proton motive force undergo autolysis, providing an additional free source of nutrients for the colony.^67^

### Fratricide behaviour during genetic transformation

In some cases, bacteria, in response to environmental signals, can induce a death program in neighbouring cells in order to uptake their genetic material (Figure 2b). Indeed in *S. pneumoniae*, cells competent for natural genetic transformation produce toxins that will kill non-competent sisters and uptake their DNA and incorporate it into their DNA by recombination.^36, 68^ In these bacteria, competence is induced by the competence-stimulating peptide (CSP) that triggers the process of transformation by interacting with its receptor ComD. Several killing factors are involved in the lysis of pneumococcal cells. The PG hydrolase CbpD is the first component and it mediates the release of DNA from sensitive streptococci. CbpD also activates two PG hydrolases (Box 1), LytA and LytC, resulting in increased lysis of susceptible cells. The process involves also two bacteriocins (CibA and CibB) that act by forming pores in the cytoplasmic membrane.^36, 68^ In addition, lysis of the killed cells causes the release of pneumolysin, a key virulent factor that contributes to the pathogenesis of the pneumococcal disease in humans.^36^

### Autolysis in *M. xanthus* development

*M. xanthus* is a free-living Gram-negative bacterium commonly found in soil with a complex life cycle that includes vegetative growth and a developmental pathway^34^ (Figure 2c). Under nutritional stress, the majority of bacteria aggregate to form a bulging mass called fruiting body while a small number of vegetative cells (about 10%), named peripheral rods, remain undifferentiated and are located around and between fruiting bodies.^69, 70^ Within the interior of the fruiting bodies, *Mixobacteria* differentiate into spores that are then released into the environment. During the early steps of development of the fruiting bodies, up to 90% of cells undergo altruistic cell lysis releasing their content which feeds the remaining cells that will differentiate into myxospores.^34, 65^ Although *M. xanthus* contains several autolysin genes encoding for PG hydrolases (Box 1), the pathway leading to autolysis is yet not well characterized.^28, 71^ In some strains, a type II TA system, MazF/MrpC, seems to be involved in cell lysis during the fruiting body formation.^71^ Before sporulation, the degradation of MrpC by cell proteases causes the activation of the toxin MazF resulting in autolysis.^71^ However, in other *M. xanthus* strains, MazF is not essential for PCD or sporulation,^72^ therefore it appears that two parallel, potentially redundant pathways leading to PCD are present in different strains.^73^

### *Streptomyces* developmental cycle

*Streptomyces* are filamentous soil bacteria, known for the production of several bioactive compounds such as antibiotics. They have a complex developmental cycle characterized by multicellular behaviour and mycelial growth.^35, 74^ *In vitro*, their life cycle shows the formation of two differentiated structures: a vegetative substrate mycelium and a reproductive aerial mycelium.^74^ In *Streptomyces* genus cycle, a part of the mycelium dies through a highly ordered process of PCD that occurs in two phases: during development of the vegetative mycelium and before sporulation (Figure 2d).^35, 75, 76^ In solid culture, the vegetative mycelium forms when *Streptomyces* grow on the agar surface forming compartmentalized mycelium (MI, first mycelium), which originates from the germination of a dormant spore when it encounters a permissive environment. Under stress conditions, such as nutrient depletion, a part of MI cells is subjected to PCD while the remaining viable cells differentiate into multinucleated mycelium (MII, second mycelium). Multinucleated mycelium would favour rapid growth and nucleoid division, crucial for sporulation.^35^ At this stage, *Streptomyces* MII cells are subjected to a new phase of PCD. A part of vegetative mycelium dies releasing nutrients to feed the aerial mycelium. The aerial hyphae are developed from branches of the remaining viable cells of MII and rise above the surface. Simultaneously, a non-growing part of the colony synthesizes antibiotics through a secondary metabolism. Finally, the apical cells of the aerial hyphae differentiate into hydrophobic spores that can spread the bacteria in the environment.^74^

When PCD occurs, the mycelium is subjected to progressive cell disorganization coupled to DNA degradation and followed by loss of the cell wall and membrane integrity and release of the intracellular content into the environment.^76^ It has been observed that proteins involved in the cellular disassembly, such as hydrolases, proteases, and enzymes implicated in membrane degradation, are produced during PCD in *Streptomyces*.^77^ The biological significance of PCD in *Streptomyces* colonies is still unclear, but it is probable that PCD is necessary to provide nutrients for further developmental processes such as sporulation and the production of antibiotics to protect the colony from neighbouring microorganisms. It has been also speculated that in these microorganisms PCD may be involved in competence, a process through which DNA fragments are incorporated in cells by transformation followed by recombination events obtaining a bundle of variable spores.^35^

Recently, it has been shown that PCD can be induced in *Streptomyces lividans* and *S. coelicolor* through a type II TA system belonging to the YefM/YoeB subfamily with high similarity to the one described in *E. coli* YefM/YoeB.^78, 79^ As in *E. coli*, the toxin acts inhibiting translation initiation by processing the mRNA three bases downstream of the initiation codon. To date, the role of this TA system in *Streptomyces* PCD is still not clear.^78^

### Coccoid forms in *Helicobacter pylori*

The bacterium *H. pylori* is recognized as the main cause of gastric and duodenal ulcers and has a primary role in the development of gastric cancer.^80, 81^ It can switch from the normal helical morphology into a coccoid-shaped resistant form when exposed to environmental stress, such as nutrients deprivation and a non-permissive temperature. In the coccoid population, a part of the cells enters a state of low metabolic activity and stops dividing (viable but non-culturable state), as a form of temporary adaption to an incompatible environment. Under these conditions, some of the coccoid cells undergo PCD, showing electron-dense bodies – probably deriving from DNA condensation – resembling micronuclei of apoptotic eukaryotic cells.^80^ This similarity is also supported by the identification of endonucleolytic DNA cleavage in these cells.^80^ It is supposed that *H. pylori* chooses to reduce its cell density through this mechanism to preserve itself and restart cell division in a suitable environment.

## Role of PCD in Biofilms

Biofilms are structurally and dynamically complex biological systems.^82, 83^ They are multicellular sessile communities characterized by cells embedded in a self-produced matrix of extracellular polymeric substances (EPS) and interspersed with open water channels. They exist both as mono- and multi-species communities.^82, 83^ EPS include proteins, polysaccharides, and extracellular DNA (eDNA). Bacteria through biofilms can adhere to biological and non-biological surfaces, such as human tissues and medical implants. Indeed, biofilm formation has a role in several infectious diseases, including endocarditis, urinary tract and cystic fibrosis infections, and infections of artificial heart valves, joint prostheses, and catheters.^82^ Furthermore, biofilms protect bacteria from stressing conditions as well as from other microorganisms that live in the same environment. Biofilms are associated with resistance to a broad range of antimicrobial agents, contributing to resistance to antibiotic treatments.^7^ Spore-forming bacteria produce both biofilm and endospores being able to respond more swiftly to environmental stresses.^84^ Moreover, biofilm is an optimal environment for sporulation.^84^

**Figure 3:**
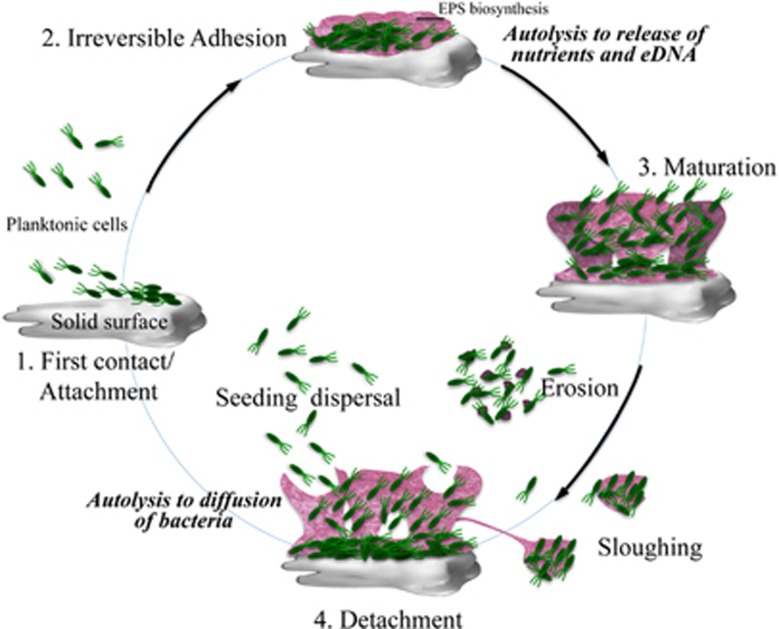
Biofilm formation and development. Initially, planktonic cells adhere to a solid surface (1), and production of extracellular polymeric substances (EPS) stabilizes the adhered colony (2). Some of the cells undergo autolysis releasing nutrients and eDNA that promote growth and maturation of the biofilm (3). Cells are dispersed from the biofilm and can colonise other sites through three mechanisms: erosion, sloughing, and seeding dispersal (4). Seeding dispersal implicates an active process of autolysis resulting in release of single bacterial cells and cavity formation

The control of bacterial cell death and lysis is supposed to be an important mechanism in cell differentiation and development of bacteria inside biofilms as well as for diffusion of bacteria into the environment.^85, 86^ For example, under starvation a part of cells inside a biofilm autolyse providing nutrients for the remaining bacteria allowing them to colonize new sites (Figure 3).^85^ To release and disperse cells from biofilm into the environment, bacteria adopt at least three different mechanisms: erosion, sloughing, and seeding.^87^ Erosion consists of the continuous release of single or small group of cells during biofilm formation. Sloughing indicates cell aggregates that are shed from biofilm, usually during its later stages of formation. Seeding dispersal is an active process in which a large number of individual cells are released from hollow cavities formed inside the biofilm.^87^ In addition, eDNA, an essential component of the biofilm matrix, is released during biofilm development by cell lysis of a part of the bacterial population. eDNA contributes to the stability and development of the biofilm by binding to other EPS biopolymers. Moreover, through an acid–base interaction, it promotes adhesion between cells and between cells and surfaces.^88, 89, 90, 91^ Several cell lysis factors such as autolysins and prophages contribute to eDNA release.^90, 91^ Extracellular DNA release is mediated by QS mechanisms in both Gram-negative and Gram-positive bacteria.^90, 91^ In the Gram-negative *P. aeruginosa*, QS molecules control the production of cell lysis factors such as prophages and phenazines (through the production of reactive oxygen species) that induce cell lysis causing eDNA release.^90^ In the Gram-positive *S. epidermidis*, the *agr* QS system regulates the expression of the autolysin AtlE, resulting in cell lysis and eDNA release.^92^

PCD has been shown to occur in biofilm in both Gram-positives and Gram-negatives through a number of mechanisms.^53, 54, 86, 93, 94, 95, 96, 97^

In *S*. *aureus*, *cidABC* and *lrgAB* operons regulate autolysis and eDNA release by controlling PG hydrolase activity through CidA and LrgA, proteins analogous to bacteriophage holins and antiholins.^86, 93^

*P. aeruginosa* is involved in chronic infections of the respiratory tract such as cystic fibrosis, a notable biofilm-based disease.^98, 99, 100^ In *P. aeruginosa*, biofilm develops in a multi-step cellular cycle that is initiated by the attachment of free cells to a surface, followed by formation and maturation of multicellular structures (microcolonies), and finally by an active dispersal mechanism that spreads bacteria resulting in occupation of new surfaces. At this stage, cell death has been observed in a subpopulation of cells within the microcolonies.^96, 101^ Dispersal and cell death events have been associated with the conversion of a genomic prophage to an infective lytic form.^96^ The action of lytic phage is correlated with the appearance inside microcolonies of phenotypic and functional variants, through transfer of genetic material, that spread and colonize new surfaces.^101^ Finally, in biofilm formation by *Streptococcus mutans*^95^ a QS system activates a death programme. Indeed, in this species when high density is reached a pherormone known as CSP is produced that induces upregulation of an autolysis effector, the CipB bacteriocin.

## Conclusions

Similarly to eukaryotes, PCD in bacteria is a complex and regulated process that contributes to multiple aspects of bacterial communities' survival, differentiation, and spreading. The similarities observed between cell death systems of animals, plants, and bacteria suggests a common origin and presumably is a consequence of the endosymbiotic acquisition of bacteria by eukaryotes.^3, 102, 103, 104^ According to this theory, aerobic and phototrophic bacteria, ingested by preeukaryotic cells, have evolved into mitochondria and chloroplasts, respectively. As previously reported, *S. aureus* CidA and LrgA proteins, located in the cytoplasmic membrane, exhibit bacteriophage holin–antiholin-like properties.^86^ The primary components of PCD regulatory control in animals, the mitochondria-associated Bax/Bcl-2 family of proteins,^1, 105, 106, 107^ also display molecular and functional similarity to holin/antiholin proteins supporting this theory.^86^ According to this idea, different organisms would use common strategies to control PCD and supposedly unlinked proteins could be evolutionarily related.^86, 102^ However, although phylogenetically conserved PCD-related genes are clearly present in Eukarya branch of the tree of life,^3^ no evidence for such a presence exists in the Bacteria and Archaea branches. Furthermore, the presence of these genes or even their partial similarity with some eukaryote genes could be attributable to phenomena, such as genetic convergence or horizontal gene transfer. A thorough description of the evolution of PCD in bacteria is, however, beyond the scope of this review.

Studies on bacterial PCD are of great interest as it can be exploited to develop efficient alternative therapeutic strategies against bacteria resistant to current antibiotics.^108^ In fact, antimicrobial resistance in bacteria is on the rise and represents a worldwide emergency, and the number of antibiotics that retain activity against several serious pathogens is limited.^109, 110^ This problem has been further amplified by the shortage of new molecules and requires novel approaches to the problem. Promising results have been obtained exploiting TA systems as antibacterial strategies via artificial PCD activation. Cell death is obtained by disruption or arrest of TA system formation or increased degradation of antitoxin.^19, 111^

However, it should also be kept in mind that PCD induction could be damaging to the host when it is coupled with the release of extracellular products. Indeed, in some cases the activation of PCD can induce an increase of extracellular products that favour the pathogens.^108^

Finally, various studies indicate QS system as an attractive alternative approach to antibiotic therapy.^49, 112, 113^ In fact, QS systems are involved in the regulation of virulence factors in bacteria, and their inhibition could increase the susceptibility of the pathogens to the host defences.

Additional investigations – such as *in vivo* studies – are required to evaluate the real potentiality of these new therapies.

## Peptidoglycan hydrolases

The family of peptidoglycan (PG) hydrolases is composed of a large heterogeneous group of enzymes that assembly, modify and degrade PG, the major component of the cell envelope whose integrity is essential to bacterial survival.^65, 114^ PG hydrolases are present in eukaryotic and prokaryotic organisms as well as in phages. In animals and plants, they are involved in the defence against bacterial infections as a part of the innate immunity.^115^ In phages, they contribute to the mechanism of infection of bacteria.^63, 64^

In bacteria, they are implicated in several physiological functions: cell wall turnover, cell separation, formation of flagella and pili, sporulation, and spore germination. Moreover, they are essential for the bacterial population being involved in developmental lysis, biofilm formation and autolysis in: genetic transformation, unfavourable growth conditions, and under exposure of antibiotics.^7, 33, 65^

PG hydrolases are classified on the specificity of their cleavage sites.^65^ PG amidases, cleave the amide bond between *N*-acetyl muramic acid and L-alanine. PG peptidases cleave peptide bonds within the peptides (endopeptidases) or remove C-terminal amino acids (carboxypeptidases). Both are further classified in DD-, LD- and DL-peptidases depending on the amino acids involved in the cleavage. The PG hydolases family also includes a group of enzymes (*N*-acetylmuramidases, lysozymes and lytic transglycosylases) that cleave the glycosidic bond between *N*-acetyl muramic acid and *N*-acetyl glucosamine. Finally, *N*-acetylglucosaminidases hydrolyse the glycosidic bond between *N*-acetylglucosamine and the neighbouring monosaccharides. This last enzyme is less specific and is involved in cleavage within oligosaccharides' different from PG.

Figure Box. Schematic representation of Gram-positive and Gram-negative bacterial cell envelopes showing the cleavage specificities of PG hydrolases. PG amidases (green arrows); Endopeptidases (red arrows); Carboxypeptidases (light blue arrows); *N*-acetylmuramidases (blue arrows); *N*-acetylglucosaminidases (magenta arrows) mDAP: *meso*-diaminopimelic acid.

## Abbreviations

PCD: programmed cell death
PG: peptidoglycan
TA: toxin–antitoxin
QS: quorum sensing
